# Pfadabhängigkeit, Bifurkationspunkte und die Rolle der Soziologie. Ein soziologischer Deutungsversuch der Corona-Krise

**DOI:** 10.1007/s11609-020-00418-2

**Published:** 2020-11-18

**Authors:** Hartmut Rosa

**Affiliations:** Allgemeine und theoretische Soziologie, Friedrich-Schiller-Universität Jena, Carl-Zeiss-Straße 3, 07743 Jena, Deutschland

**Keywords:** SARS-CoV‑2, COVID-19, Entschleunigung, Systemtheorie, Pfadabhängigkeit, Paradigmenwechsel, Best account, SARS-CoV‑2, COVID-19, Deceleration, System theory, Path dependency, Paradigm change, Best account, SARS-CoV‑2, COVID-19, Décélération, Théorie des systèmes, Dépendance au sentier, Changement de paradigme, Best account

## Abstract

Der Beitrag versucht zunächst, das Wesen und die Erscheinungsform der Corona-Krise mit beschleunigungstheoretischen Mitteln genauer zu bestimmen. Zentral ist dabei die Diagnose einer politisch herbeigeführten, objektiv messbaren gesellschaftlichen Entschleunigung. Auf dieser Basis werden dann im zweiten Schritt in Auseinandersetzung mit der Systemtheorie einerseits und mit neomarxistischen Ansätzen andererseits die gesellschaftstheoretischen Konsequenzen gezogen. Der Fokus liegt dabei auf dem Argument, dass sich die politische Reaktion auf das SARS-CoV-2-Virus weder nach den Prinzipien funktionaler Differenzierung noch aus der Logik der Herrschaftssicherung oder der Kapitalakkumulation erklären lässt. Im Anschluss daran arbeitet der Beitrag heraus, welche Möglichkeiten sich in der Krisenlage für einen gesellschaftlichen Pfad- und Systemwechsel ergeben und welche Rolle die Soziologie als wissenschaftliche Disziplin in dieser historischen Lage spielen kann und spielen sollte.

## Abstract (en)

The contribution begins by attempting to specify the corona crisis’ essence and form by means of acceleration theory. In so doing, the diagnosis of a politically induced, objectively measurable deceleration is pivotal. In a second step, the social theoretical conclusions from this diagnosis are drawn in contrast to system theory on the one hand, and neomarxist approaches on the other. The focus is on the argument that the political reaction to SARS-CoV‑2 can neither be explained by reference to the principles of functional differentiation nor through the logic of securing political power or through capital accumulation. Following this line of thought, the article maps out the possibilities created by the crisis for societal path and system change, and outlines the role that sociology as a scientific discipline can and should play in this historical situation.

## Résumé

Cet article tente tout d’abord de déterminer plus précisément la nature et les manifestations de la crise du coronavirus à l’aide de la théorie de l’accélération. Dans ce cadre, le constat d’une décélération sociale provoquée par la politique et mesurable objectivement occupe une place centrale. De là, on tire dans un second temps les conséquences de la crise en termes de théorie sociale en la confrontant d’une part à la théorie des systèmes, d’autre part aux approches néo-marxistes. Ce faisant, l’accent est mis sur l’argument selon lequel la réaction politique au virus SARS-CoV‑2 ne s’explique ni par les principes de la différenciation fonctionnelle ni par la logique du maintien de la domination ou de l’accumulation du capital. Ensuite, cet article met en évidence les possibilités de changement de sentier ou de système social que recèle cette situation de crise et le rôle que la sociologie peut et devrait jouer en tant que discipline scientifique dans cette situation historique.

## Einleitung

Auch wenn es für die Perspektive einer Eule der Minerva, die ihre Erkenntnis erst in der historischen Rückschau gewinnt, definitiv zu früh ist, weil die durch das Auftreten des SARS-CoV-2-Virus ausgelöste soziale, politische und ökonomische Krise noch keineswegs gelöst oder überwunden ist, stimmen doch nahezu alle sozialwissenschaftlichen Beobachter darin überein, dass diese Krise einen markanten, globalen Einschnitt in die vorherrschenden sozioökonomischen und politischen Interaktionsmuster und Prozessketten verursachte und deshalb eine historische Zäsur signalisiert. Mit „Krise“ meine ich im Folgenden nicht einfach nur das Auftreten des Virus und dessen gesundheitliche Folgen, sondern ebenso sehr die darauf reagierenden politischen Maßnahmen und ihre rechtlichen, sozialen und ökonomischen Konsequenzen.

Ich möchte zunächst das Wesen und die Erscheinungsform dieser Krise mit beschleunigungstheoretischen Mitteln genauer bestimmen, wobei der Fokus naturgemäß nicht auf der virologisch-medizinischen, sondern auf der soziopolitischen Seite des Phänomens liegen wird. Im Anschluss daran werde ich herausarbeiten, welche Möglichkeiten für einen gesellschaftlichen Pfad- und Systemwechsel sich aus meiner Sicht daraus ergeben – und *dass* sich *realiter* solche Möglichkeiten ergeben – und welche Rolle die Soziologie als wissenschaftliche Disziplin in dieser historischen Lage spielen kann und spielen sollte. Dem letzteren Gesichtspunkt – und der Frage, mit welcher Methodologie die Soziologie die ihr zugedachte Aufgabe am besten bewältigen kann – gilt mein Hauptaugenmerk.

## Bestimmung der Krise: Die COVID-19-Zäsur

Es kann kein Zweifel daran bestehen, dass das Virus SARS-CoV‑2 zunächst einmal und unmittelbar eine gewaltige menschliche Tragödie bedeutet, indem es durch Krankheit und Tod und durch die infolge der Pandemie entstandene Armut und Not millionenfaches Leid über die Welt brachte und bringt. Darüber hinaus erzeugt es im Verbund mit den ergriffenen gesundheitspolitischen Maßnahmen massive ökonomische Probleme für ein globales Wirtschaftssystem, das ohne Zweifel auch schon vor dieser Krise auf tönernen Füßen stand. Deshalb birgt es auch gravierende politische Gefahren, wie sie sich etwa in der Umbildung demokratischer zu autoritären Strukturen, in der Außerkraftsetzung elementarer Bürgerrechte und in der Rückkehr von zum Teil primitivsten Formen nationalstaatlicher Schließung zu erkennen geben. Ich will diese Seite der Ereignisse jedoch zunächst beiseite lassen und aus einer nüchtern-gesellschaftstheoretischen Perspektive danach fragen, welche systemisch-strukturelle Bedeutung der aktuellen Krise zuzumessen ist.

Meine These lautet, dass wir es in der Tat (und anders, als es aus einer systemtheoretischen oder orthodox kapitalismustheoretischen Sicht erscheinen mag) mit einer gewaltigen strukturellen Erschütterung und *Destabilisierung* des global dominanten Sozial- und Gesellschaftssystems zu tun haben. Denn dieses System ist nach meiner Überzeugung durch einen spezifischen Stabilisierungsmodus gekennzeichnet, den ich in vielen Publikationen als *Modus dynamischer Stabilisierung* zu identifizieren versucht habe.

Dynamische Stabilisierung meint, dass die Basisinstitutionen der Gesellschaft – die kapitalistische Organisation der Wirtschaft, die demokratisch-repräsentative Organisation der Politik, die forschungsorientierte Arbeit der Wissenschaft, die sozialstaatliche Organisation der Wohlfahrt sowie die Bildungsinstitutionen und der Kunstbetrieb – sich nur im Modus der Steigerung zu reproduzieren und zu erhalten vermögen, dass sie mithin also systematisch auf (ökonomisches) Wachstum, auf (technische und kulturelle) Beschleunigung sowie auf politische Aktivierung und, damit verknüpft, auf beständige Innovationsleistungen angewiesen sind, um ihren Status quo zu stabilisieren und ihre Struktur zu erhalten. Dies führt im Ergebnis zu einer genuin eskalatorischen Tendenz, welche die Steigerungsleistungen in ihrer Substanz in vielen Bereichen (etwa in Form der Zunahme des Verkehrsaufkommens, des Verbrauchs von Rohstoffen und der Produktion von Gütern) über viele Dezennien hinweg nicht linear, sondern exponentiell anwachsen lässt.^1^

Eine moderne Gesellschaft, so lässt sich diese Einsicht zusammenfassen, ist dadurch gekennzeichnet, dass sie sich immerzu steigern und dynamisieren muss, um sich zu erhalten, und mithin also dadurch, dass ihre kinetische Energie notwendig steigt. Die realen physisch-ökonomischen Konsequenzen dieses Modus macht jeder Blick auf die Datenlage, vor allem aber auf das *materielle Bewegungsprofil* der Erde klar: Seit dem 18. Jahrhundert befindet sich die Welt (wenn wir sie gleichsam als Ganzes mit einem Blick von außen betrachten) in einem – gewiss uneinheitlichen, ungleichzeitigen und oft gewaltförmig verlaufenden – Prozess der Dynamisierung, der sie buchstäblich in immer schnellere Bewegung versetzt (hat). Seit 1800 haben sich die weltweite Produktion von Gütern und Dienstleistungen, die physische Bewegung des Erdreiches und der technisch vermittelte Stoffwechselprozess mit der Natur, die Anzahl der Fahrzeuge aller Art und der Menschen und Güter, die damit in Bewegung gebracht werden, ebenso wie der Ausstoß an Gift- und Schadstoffen ununterbrochen vermehrt, zum Teil um das Hundert- und Tausendfache. Blickt man auf das Bewegungsprofil der Erde, also auf die zu einem Zeitpunkt durchschnittlich bewegte *Masse* an Menschen, Rohstoffen und Gütern und auf die Geschwindigkeit dieser Bewegung, dann erhält man eine exponentielle Wachstumskurve, die – von kleinen Schwankungen abgesehen – praktisch keine Pausen oder Grenzen kennt. Sicherlich haben wirtschaftliche Rezessionen oder Kriege für kurze Zeiträume und meist lokal begrenzt die Geschwindigkeit der Produktion und der Bewegung vorübergehend gedrosselt, aber dies erzeugte fast immer neue Opportunitäten für weiteres Wachstum und Beschleunigung. Wie Paul Virilio (1980, 1994) in seinen Arbeiten gezeigt hat, haben gerade Kriege nahezu invariant als machtvolle Motoren der Beschleunigung und der Mobilmachung gedient.

Wenn ich einen Systembruch infolge der weltweiten Reaktionen auf SARS-CoV‑2 postuliere, so zeigt er sich exakt im Blick auf dieses physische Bewegungsprofil der Erde und damit geradewegs in einer naturwissenschaftlichen Kriterien standhaltenden Perspektive: Nach mehr als zwei Jahrhunderten nahezu ungebrochener Beschleunigung und Dynamisierung bremst die Welt plötzlich ab, sie wird in ihrer materiell-physischen Bewegung abrupt und radikal langsamer.^2^ Es hat den Anschein, als hätte jemand gigantische Bremsen an die Räder der Produktion, des Transports, aber auch des sozialen und kulturellen Lebens angelegt. Dieser Befund hat auch dann Bestand, wenn man sich vor Augen führt, dass keineswegs alle Bereiche des Transports, der Produktion und des sozialen Lebens betroffen waren, dass in einigen Branchen insbesondere des Gesundheitswesens, aber zum Teil auch der Produktion etwa von Grundnahrungsmitteln, digitalen Endgeräten oder sogar von Toilettenpapier, signifikante Wachstums- und Beschleunigungsprozesse zu konstatieren sind und es insgesamt zu einer massiven Steigerung digitaler Produktions- und Kommunikationsprozesse gekommen ist. Tatsächlich halte ich die scharfe Entkopplung zwischen einem sich verlangsamenden physisch-realen Verkehr und einer sich beschleunigenden digitalen Zirkulation, Kommunikation und Produktion für eine der auch strukturell bedeutsamsten Nebenfolgen der aktuellen Krise. Sie manifestiert ansatzweise das, was Virilio mit seiner Vision eines „rasenden Stillstandes“ als dystopischen zivilisatorischen Endzustand schon um 1990 postulierte: eine Gesellschaft, in der der physische Bewegungsradius der Menschen sich immer weiter reduziert, während das „Rauschen“ und die Geschwindigkeit der digitalen Ströme immer weiter zunimmt (Virilio 1992). Dass der erste Teil dieser Vision – die partielle „Stillstellung“ der Menschenströme und die Verlangsamung des physischen Bewegungsprofils der Erde – bisher rein utopisch bzw. dystopisch schien, sich nun aber realiter beobachten lässt, kann als Indikator für das Neue dieser Situation dienen. Die Verlangsamung oder, ja, die *Zwangsentschleunigung*, zeigt sich in allen Lebens- und Mobilitätsbereichen, am spektakulärsten sicherlich im Flugverkehr: Bis zu 85 % der Flüge wurden gestrichen. Der Himmel über Europa wurde in wenigen Tagen leergeräumt; wer hätte das bis vor einem Jahr für möglich gehalten? Entschleunigung stellt also ein hartes, global beobachtbares soziales und materielles Faktum dar; sie zu diagnostizieren ist keine rückwärtsgewandte, nostalgische oder gar reaktionäre Fantasie, wie einige Kritiker dieser Diagnose behaupten. Insbesondere in der Welt der Produktion und des Verkehrs lassen sich massive globale Reduktionen von teilweise über 80 % des Volumens beobachten, und auch der Kultur- und Bildungsbetrieb ist (oder war) vielerorts fast völlig zum Erliegen gekommen (Dambeck und Tack 2020).

Kein Treibhauseffekt, keine Hitzewelle, kein Tornado und auch keine „Fridays for Future“-Bewegung und schon gar keine Klimakonferenz erzielte davor jemals irgendeinen nennenswerten Verlangsamungseffekt. Als zweites bedeutsames Faktum muss dabei gelten, dass die Verlangsamung *nicht* der unmittelbare Effekt des Virus selbst ist, sondern eine Folge politischer Entscheidungen und politischen Handelns durch größtenteils demokratisch gewählte Regierungen, die unter Billigung der Parlamente und mit der mehrheitlichen Zustimmung der Regierten die Bremsen anlegten. Nicht das Virus holte die Flugzeuge vom Himmel und stoppte den Spielbetrieb in den Fußball-Ligen, sondern das staatliche politische Handeln. Warum ist das bemerkenswert? Weil dieselben politischen Akteure sich seit nunmehr fünfzig Jahren zwar in vielerlei Hinsicht willens, aber vollkommen unfähig zeigen, gegen das (insbesondere durch die Kapitalakkumulation getriebene) Räderwerk der Beschleunigung bzw. gegen seine ökologisch schädlichen Nebenfolgen Wirkungsvolles auszurichten. Seit dem ersten Bericht des „Club of Rome“ (Meadows et al. 1972) entstanden viele Bücher, Parteien und Bewegungen, die gegen die Steigerungslogik und den durch sie angetriebenen Ressourcenverbrauch anschrieben, anschrien und ankämpften; eine Klimakonferenz jagte die nächste, eine politische Erklärung folgte der anderen, aber im Grunde änderte sich: nichts. Die Zahl der produzierten Autos und der geteerten Straßen, die Zahl und Tonnage der Lastkraftwagen, die Zahl und das Volumen der Containerschiffe und der Kreuzfahrtschiffe, der Passagiere im Nah- und Fernverkehr, der Hochgeschwindigkeitszüge: sie alle stiegen rapide nicht nur in Asien, sondern auch noch in Europa. Und im Flugverkehr als der jüngsten physischen Mobilisierungssphäre ergab sich allerorten ein exponentielles Wachstum der Flugzeug‑, Flug- und Passagierzahlen. Die Antriebsmechanismen schienen vollkommen immun gegen jede Art von Wachstums- und Beschleunigungskritik, und auch die immer deutlicher werdenden Zeichen und Folgen der Klimakrise und die auf sie reagierenden politischen Beschlüsse und Erklärungen prallten ab an der stahlharten Steigerungslogik moderner Gesellschaften und kapitalistischer Wirtschaften. Doch im April 2020 steht ein erheblicher Teil der Räder plötzlich still, ist zumindest die Antriebskraft deutlich reduziert. Dass selbstredend *nicht* die ganze Welt angehalten wurde, tut dem Faktum keinen Abbruch, dass erstmals in der Geschichte der Moderne die physisch-materielle Bewegung auf der Erde durch bewusstes politisches Handeln (und nicht als unintendierte Nebenfolge ökonomischer oder militärischer Krisen) verlangsamt wurde. Und auch wenn etwa das Reduktionsvolumen der täglichen globalen CO_2_-Emissionen „nur“ 17 % betrug: Wie ein Autor*innenteam um Corinne Le Quéré und Rob Jackson (2020) in einem Beitrag in der Zeitschrift „Nature Climate Change“ aus den Daten des „Global Carbon Project“ berechnet hat, handelt es sich dabei immerhin um mehr als eine Milliarde Tonnen CO_2_-Emissionen, die als unmittelbare Folge bewusst verlangsamenden Regierungshandelns eingespart wurden, während im Vergleich dazu die Finanzmarktkrise von 2008, gegen die alle nur erdenklichen politischen Maßnahmen unternommen wurden, das Bewegungsprofil nur schwach beeinflusste – was sich 2009 in einer vorübergehenden Reduktion der globalen CO_2_-Emissionen um gerade einmal 1,4 % niederschlug, auf die allerdings im Jahr 2010 bereits wieder ein Anstieg um 5,1 % folgte.^3^

## Gesellschaftstheoretische Konsequenzen

Um es noch einmal zu sagen: Dieser gewaltige Einschnitt in die Handlungsroutinen und Prozessketten der Moderne, der die routinierten Handlungslogiken und Interaktionsmuster in weiten Gesellschaftsbereichen bis hinein in die Alltagspraktiken der Menschen gravierend verändert hat, war und ist das Ergebnis gezielten und planvollen politischen Handelns. Dies erscheint als überraschend vor dem Hintergrund der Tatsache, dass sich eben dieses staatliche Handeln als fast vollkommen machtlos präsentiert und erwiesen hat – sowohl angesichts der globalen Klimakrise, welche Umfragen zufolge von den deutschen Wählern 2019 als das mit Abstand drängendste politische Problem empfunden wurde,^4^ als auch angesichts der als immer gravierender erfahrenen globalen Ungleichheit und Ungerechtigkeit bei der (nationalen und globalen) Einkommens- und Vermögensentwicklung. Gegenüber der Eigenlogik der (Finanz‑)Märkte, der Technikentwicklung und der Konsumgewohnheiten schien politisches Handeln nahezu wirkungslos, gegen das „stahlharte Gehäuse“ der kapitalistischen Steigerungslogik schien nichts auszurichten.

Tatsächlich gibt es zwei einflussreiche gesellschaftstheoretische Ansätze, welche diese Machtlosigkeit scheinbar schlüssig zu erklären vermochten: die Systemtheorie und der herrschaftstheoretisch-strukturdeterministische (Neo‑)Marxismus. Beide Ansätze erweisen sich in ihrer Erklärungskraft angesichts der gesellschaftlichen Reaktionen auf die Corona-Krise als limitiert; und diese Unzulänglichkeit, so möchte ich zeigen, ist in beiden Fällen systematischer Natur.

Die Grundlage der an Luhmann ausgerichteten Systemtheorie, wie sie etwa Armin Nassehi vertritt, bildet die Überzeugung, dass die funktionale Differenzierung das Kernprinzip und die Essenz moderner Gesellschaften beschreibt: Durch die Umstellung gesellschaftlicher Strukturierung vom „alteuropäischen“ Prinzip der ständisch-hierarchischen Ordnung auf einen Modus funktionaler systemischer Ausdifferenzierung hat die moderne Gesellschaft ihre Spitze und ihr Zentrum verloren; sie ist polykontextural und damit „komplex“ geworden, wie das unaufhörlich wiederholte Mantra dieser Theorierichtung lautet. Die ausdifferenzierten Funktionssysteme Wirtschaft, Wissenschaft, Politik, Recht, Religion etc. können einander allenfalls noch irritieren oder auf problematische Weise „agitieren“, aber keinesfalls gezielt steuern – weil jedes Funktionssystem eigensinnig, um nicht zu sagen: starrsinnig, seinem je eigenen operativen Code folgt. Daher gibt es keine geteilte Perspektive mehr auf die Welt, keine gemeinsamen Problemdefinitionen, kein „konzertiertes Handeln“.

Die Folgen solcher funktionalen Differenzierung, so die systemtheoretische Überzeugung, zeigen sich etwa an der Klimakrise: Sie mag im politischen System noch so aufgeregt als „Krise“ kommuniziert werden, für die Wirtschaft wird sie erst und nur relevant, wenn es sich im Blick auf die Zahlungsfähigkeit und -willigkeit in irgendeiner Weise „rechnet“. Und *wie* es sich dann jeweils rechnet, lässt sich politisch nicht kontrollieren (so schon Luhmann 1986). Und in der Tat, die Wirklichkeit schien dieser Theorie recht zu geben: Der weltweite Verbrauch an nicht-erneuerbaren Rohstoffen, die Produktion giftiger Abfallstoffe und von Treibhausgasemissionen stiegen Jahr für Jahr in einer Weise, die sich als immun gegenüber allen Klimakonferenzen, politischen Beschlüssen oder auch sozialen Protesten erwies – dagegen konnten auch Millionen von „Fridays for Future“-Kids nichts ausrichten, die von Systemtheoretiker*innen nur mild belächelt werden. Das gleiche Bild zeigt sich im Blick auf globale Verteilungsströme: Die Kluft zwischen arm und reich wächst allem Protest und aller Kritik zum Trotz unaufhörlich; acht Männer verfügen zusammen über die gleiche Vermögenssumme wie die knapp vier Milliarden Menschen, die zur ärmeren Hälfte der Weltbevölkerung gehören.^5^

Dann aber, im März 2020, erhält dieses Bild plötzlich einen Riss: Die Gesellschaft scheint eine Spitze und ein Zentrum zu haben; von einem Tag auf den anderen wird der Staat zum zentralen Akteur und ordnet alle Gesichtspunkte dem einen Ziel – dem Kampf gegen das Corona-Virus – unter. Ohne Gewalt und Blutvergießen schließt er die Landesgrenzen, holt er 85 % der Flugzeuge vom Himmel, schließt Schulen und Universitäten, hält Fußballligen an, greift massiv in die Produktion ein. Und es ist keineswegs nur ein destruktiver Akt des Anhaltens: Er initiiert auch Forschungsprozesse, aktiviert Gesundheitssysteme und erzwingt zielgerichtet die Herstellung spezifischer Güter, Medikamente oder Werkzeuge zur Eindämmung der Pandemie. Im vielleicht marktradikalsten Land der Welt, den Vereinigten Staaten, ordnet der Präsident einen seiner mächtigsten Produzenten, General Motors, kurzerhand an, anstelle von Automobilen Beatmungsgeräte zu produzieren. Diese radikale Außerkraftsetzung der Logik funktionaler Differenzierung, oder vielmehr: ihre Unterordnung unter ein konzentriertes und konzertiertes und darüber hinaus, zur großen Überraschung der Systemtheoretiker*innen, auch noch *effizientes* zielgerichtetes politisches Handeln, legt den Verdacht nahe, dass die systemtheoretische Sicht auf die moderne Gesellschaft unterkomplex war: „Es geschieht gerade etwas, von dem wir immer gesagt haben: Das geht nicht“, konstatierte Armin Nassehi (2020a) so verblüfft wie folgerichtig im April 2020 in einem Interview mit dem „Spiegel“.

Was Nassehi damit meint, wird deutlich, wenn man sein neuestes, unmittelbar vor der Corona-Krise erschienenes Buch *Das große Nein* liest. Seine These dort lautet, dass es in der modernen Gesellschaft *strukturell* unmöglich sei, auch nur einen gesamtgesellschaftlichen *Vetospieler* zu installieren (der etwa die Treibhausgasemissionen anhalten könnte). Eben dies generiere kontinuierlich sozialen Protest.

Zur Grunderfahrung der Moderne gehört, dass die bloße Aufklärung über Missverhältnisse oder die bloße Erkenntnis über das, was zu tun sei […], sich nicht einfach in Handlungen umsetzen lässt. Das ist eine demütigende Erfahrung. Sie widerspricht letztlich der Idee intentionaler Handlungskonzepte. Aber es ist […] vielleicht die zentrale Erkenntnis einer sozialwissenschaftlich informierten Selbstbeobachtung der modernen Gesellschaft […]. Gesellschaftliche Modernität ähnelt dem, was man im politischen Bereich Gewaltenteilung nennt. Es ist ein evolutionärer Prozess, dessen Gesamtrichtung dahin geht, dass es Instanzen, die alles zusammenführen und in einem Prinzip aufheben, nicht mehr geben kann. […] Soziologisch gesprochen: *Diese Struktur der Differenzierung* […] *suspendiert die Position des Vetospielers, sie schließt Positionen aus, die in der Lage wären, das Gesamtsystem zu führen, zu stoppen, zu bestimmen.* […] Alle Strukturen sind darauf ausgerichtet, dass die Gesellschaft nicht aus einem Guss handeln kann und *dass sich jegliche Initiative an der Perspektivendifferenz der Gesellschaft, an den Autonomisierungen ihrer Funktionen und nicht zuletzt an der Nicht-Erreichbarkeit des konkreten Verhaltens von Individuen bricht*. (Nassehi 2020b, S. 68 f.; erste Hervorh. i. Orig., zweite Hervorh. H. R.)

Gerade weil Nassehis These der „demütigenden Erfahrung“ einer handlungsunfähigen gesellschaftlichen Ohnmacht angesichts manifester globaler Probleme in den vergangenen Jahrzehnten eine hohe Plausibilität für sich beanspruchen konnte, habe ich in meinen ersten Äußerungen zu den Folgen der Corona-Krise (gegen die Einwände etwa Klaus Dörres [2020a, S. 31]) darauf insistiert, dass die überraschende Effizienz und Stringenz des Handelns in der Krise eine spektakuläre politische Selbstwirksamkeitserfahrung für die Gesellschaft bedeute – eine Erfahrung, die ihr vor Augen führt, dass sie sehr wohl über ein Organ und einen Modus verfügt, gesellschaftliche Prozesse anzuhalten und soziales Handeln (bis in das leibliche Verhalten der Einzelnen hinein) zielgerichtet zu bündeln.

Anders als Nassehi also annimmt, offenbart sich damit und darin, dass die moderne Gesellschaft unter der Logik funktionaler und systemischer Differenzierung – hinter dem von der Systemtheorie für fundamental gehaltenen Strukturprinzip – durchaus weiterhin über einen (nicht einfach nur alteuropäisch überholten) Sinn für ihre Einheit und mit ihm auch über eine politische Handlungsfähigkeit verfügt, welche ihr in Krisenzeiten im Staat eine schlagkräftige Spitze und ein Zentrum zu geben vermag. Die Vorstellung, die Politik bilde einfach nur *ein* System neben allen anderen, erweist sich im Frühjahr 2020 insofern als eine kategoriale Fehleinschätzung. Was sich vielmehr zeigt, ist, dass die Aufrechterhaltung der funktionalen Differenzierung, das ungehinderte subsystemische Prozessieren, erstens, eine staatlich geschaffene und garantierte gesellschaftliche „Einrichtung“ ist, die, zweitens, nur solange unangefochtenen Bestand hat, wie sie sich in den Augen der politisch wirkmächtigen Akteure (und dazu zählt in halbwegs funktionierenden Demokratien natürlich auch der *demos*) sowie der dominanten Legitimationsdiskurse als wünschenswert bzw. „funktional“ erweist.

Die Verblüffung der Systemtheorie wiederum quittieren die Protagonisten jener anderen wirkmächtigen gesellschaftstheoretischen Strömung, die ich als herrschaftstheoretisch-strukturdeterministisch bezeichnen möchte und der ich hier neben Autor*innen wie Nancy Fraser oder David Harvey, ungeachtet der zweifellos großen Unterschiede zwischen ihnen, meine beiden Diskussionspartner Klaus Dörre und Stephan Lessenich zurechne, mit einem müden „Wir haben es Euch ja immer gesagt!“-Lächeln. Unter dieser etwas umständlichen Bezeichnung fasse ich gesellschaftstheoretische Ansätze zusammen, die strukturanalytisch und sozialontologisch auf eine antagonistische Oben-unten-Unterscheidung fixiert sind, welche die Perspektive auf die Möglichkeiten staatlichen Handelns prädeterminiert. Sie beziehen ihre Inspiration in der Regel aus dem klassentheoretisch argumentierenden Neomarxismus, zum Teil aber auch aus poststrukturalistischen und gouvernementalitätstheoretischen Entwürfen. Für die Vertreter*innen dieser Denkrichtung bestand nie ein Zweifel daran, dass der Staat ein überaus wirkmächtiger zentraler Akteur ist – ein Akteur, der im Interesse der Kapitalakkumulation bzw. der herrschenden Klassen agiert und reagiert und dabei jederzeit in die operative Autonomie der Teilsysteme einzugreifen vermag. Dass der Staat seine Ziele notfalls mit Gewalt in allen Subsystemen durchsetzen kann und dies auch immer wieder tut, scheint für Dörre (2020b) beispielsweise eine Binsenweisheit zu sein. Und in der Tat können die Vertreter dieser Position etwa auf die massiven staatlichen Eingriffe zur Rettung von Banken und Unternehmen in und nach der Finanzkrise von 2008/09 verweisen, aber auch auf die stets kapitalfreundliche, in den letzten Jahrzehnten meist neoliberale Steuerung von Entwicklungen etwa im Gesundheits- oder Bildungssektor oder auf die auch gewaltförmige Regulierung von Migrationsströmen. Auch in der Finanzkrise war das Kriterium der „Systemrelevanz“ das entscheidende für die Begründung und Rechtfertigung staatlicher Eingriffe: Banken und Finanzunternehmen wurden gerettet, weil sie systemrelevant waren. Es drohe andernfalls der Zusammenbruch der Wirtschaftskreisläufe und des ökonomischen Systems, so lautete die Begründung für das ‚„systemwidrige‘“ politische Handeln. Für Klaus Dörre wie für Stephan Lessenich besteht daher nicht der geringste Zweifel daran, dass der machtvoll handelnde Staat das Instrument „der Herrschenden“ bzw. der herrschenden Klasse ist: Er setzt deren Interessen rigoros durch und nutzt Gelegenheiten wie die Corona-Pandemie als willkommenen Vorwand, um emanzipatorische Interessen zu unterdrücken. Für die Protagonisten dieses Denkens gilt: Was auch immer im Rahmen staatlichen Handelns geschieht, geschieht letzten Endes im (ökonomischen) Interesse der Reichen und Mächtigen, und sei es auch in deren aufgeklärtem Eigeninteresse, das sich der Notwendigkeit von Klassenkompromissen, Zugeständnissen und ideologischen Flankierungen bewusst ist. Das staatliche Handeln ist insbesondere durch die von Klassenlagen geprägten Kräfteverhältnisse bestimmt und in diesem Sinne gleichsam strukturdeterminiert.

Hier zeigt sich eine markante Kluft zwischen den oft sehr elaborierten theoretischen Entwürfen und den konkreten politischen Analysen und Diagnosen der Vertreter*innen dieser Denkströmung: Während die theoretischen Entwürfe etwa im Sinne der Regulationstheorie, der Hegemonietheorie oder der Spätkapitalismustheorie in der Regel die Eigendynamiken, Ambivalenzen und Spielräume staatlichen Handelns und politischen Aushandelns anerkennen, zeigen die politischen Analysen eine oft fatale Tendenz zur (simplifizierenden) Reduktion, nach der am Ende „sowieso“ die Reichen und Mächtigen gewinnen.^6^ Dass Staaten sich aktuell als machtvoll handelnde politische Akteure erweisen, gibt daher für diese Position keinerlei Anlass zu Optimismus, sondern wird eher im Gegenteil als Fanal zum Abbau aller Sicherungen zum Schutz vor den schrankenlosen Kapital- und Herrschaftsinteressen gefürchtet. Tatsächlich lese ich die bisherigen Stellungnahmen meiner Debattenpartner als in geradezu monomanischer Weise auf diese strukturelle Oben-unten-Perspektive fixiert. Dazu passt denn auch, dass Dörre (2020a, S. 26) meine Position (zusammen mit der aller anderen, die Hoffnung schöpfen) im Sinne des frühen Marx einer „neuen Deutschen Ideologie“ zurechnet – einer Ideologie, die übersieht, dass der Staat nur der verlängerte Arm des Kapitals ist.^7^ Ganz ebenso wie für die Systemtheorie scheitert auch für die Vertreter des herrschaftstheoretisch-strukturdeterministischen Ansatzes damit jede Hoffnung auf Veränderung oder Verbesserung an einer knallharten Strukturgesetzlichkeit.

Das Problem ist dabei nur, dass diese Strukturgesetzlichkeit in keiner Weise die weltweit erstaunlich ähnlichen Reaktionen der Nationalstaaten auf das Auftreten von SARS-CoV‑2 zu erklären vermag. Zwar ist auch jetzt „Systemrelevanz“ eines der zentralen Schlagwörter der Stunde, wenn es um politische Entscheidungen über Öffnungen und Schließungen, staatliche Aneignungen, Eingriffe und Umverteilungen angesichts der Corona-Krise geht. Doch das Wort hat im Vergleich zur Finanzkrise nun eine vollkommen andere Bedeutung: Das staatliche Handeln gilt nicht der Sicherung der Banken, der Märkte und der Kapitalakkumulation, sondern setzt deren Funktionieren und Prosperieren teilweise radikal aufs Spiel, um die Alten und die durch Vorerkrankungen Geschwächten zu schützen, die durch das Virus am meisten gefährdet sind und waren. Ganz unabhängig von der Frage, welche Schäden und Verheerungen COVID-19 wirklich anrichtet, kann als herrschende Überzeugung gelten, dass vor allem die genannten Bevölkerungsgruppen das Coronavirus zu fürchten hätten, während es bei den übrigen meist nur milde, allenfalls grippeähnliche Symptome auslöse. Das Sterben von Hochbetagten, Kranken und Schwachen, die Notwendigkeit der Triage stellen nun aber ganz offensichtlich kein Systemproblem für die Finanzmärkte und die Kapitalakkumulation dar – ganz im Gegenteil, die Pandemie verspricht geradezu, „demografische Schieflagen“ zu beseitigen und die Krankenkassen und Rentensysteme nachhaltig zu entlasten. Eine solche Sichtweise wäre selbstredend über alle Maßen zynisch, läge aber durchaus im „Herrschaftsinteresse“, sofern man dieses nur nach jener Strukturgesetzlichkeit denkt.

Was die Krise daher offenbart hat, ist das Auftreten eines Spaltes, einer Kluft zwischen dem entschlossenen staatlichen Handeln und der Kapitalakkumulation: Die Politik hat sich brachial gegen die Interessen sämtlicher Wirtschaftsbranchen und Finanzmarktlogiken durchgesetzt, und sie hat dies im Namen einer ganz anderen Art von Relevanz getan: Wenn in den Krisenmonaten von Systemrelevanz die Rede war, so war damit das biologische und soziale Leben gemeint, nicht das ökonomische System. Die Krankenpfleger- und Ärzt*innen sind (system-)relevant, nicht die Wachstumsraten und Börsendaten. Was hier aufscheint oder aufschien, war die Priorität der Reproduktionslogik über die Produktionslogik. Und das staatliche Handeln verteidigte die Erstere in dieser Lage nicht *gegen die Interessen der Beherrschten*, sondern durchaus *in ihrem Interesse*. Ganz ebenso wie Nassehi konstatieren musste, dass gerade etwas geschieht, „von dem wir immer gesagt haben: Das geht nicht“, müssten auch die Protagonisten des herrschaftstheoretisch-strukturdeterministischen Ansatzes dies eingestehen. Doch das tun sie nicht. Sie erklären den plötzlich auftretenden Struktur- und Strategiebruch, der in der gezielten Außerkraftsetzung der Antriebsprinzipien dynamischer Stabilisierung liegt, erst gar nicht, sondern beschränken sich auf die (gewiss richtige) Beobachtung, dass diese Krise vielerorts die Lage der Subalternen verschlimmere, sowie auf – wie ich gleich zeigen werde, analytisch nicht gedeckte und normativ bzw. politisch verheerende – düstere Prognosen, nach denen nun „massive Entsolidarisierungen“ und ein ökologischer Rückbau zu erwarten stünden (Dörre 2020d, Abschn. 5.2). Subsumtionslogisch wird dann das gesamte Krisengeschehen in die eine theorieleitende Narration gepackt, nach der das politische Handeln nur die Folge zeitigen kann, die Herrschenden reicher und die Verwundbaren ärmer zu machen: „In den Elendszonen sowohl des Nordens als auch des Südens finden wir bestätigt, was für Pandemien schon immer galt. Die Ungleichheit nimmt zu, und sie wird vor allem jenen schaden, denen alsbald auch noch der Teller für die Suppe fehlen könnte“, schreibt Klaus Dörre (ebd.). Und Stephan Lessenich antwortet auf die Frage, wem das beobachtbare nationale Krisenhandeln nutze: „Erst mal zahlt es auf das Konto derer ein, die von den herrschenden Verhältnissen profitieren. Und das sind Menschen wie beispielsweise ich. Aber langfristig zahlt es, wenn überhaupt, auf das Konto von ganz, ganz wenigen ein, also vielleicht von denen, die sich auch in der Krise noch wirtschaftlich bereichern können“ (Lessenich 2020). Wer dergestalt die Erkenntnis und Formulierung des realsozial und realpolitisch in Erscheinung tretenden Spalts zwischen staatlichem Handeln und Kapitalakkumulation als Ideologie zu denunzieren trachtet, erscheint mir aber als zwanghaft auf den Status quo fixiert; er oder sie betreibt, wie ich noch genauer zeigen möchte, unbeabsichtigt letztlich das Geschäft konservativer Politik und die Verteidigung der Kapitalinteressen.

Ein politisch handlungsfähiger Staat, der die Interessen der Menschen und des Lebens gegen die Kapitalinteressen vertritt: das mag gewiss nicht das politisch dominante Bild der kapitalistischen Moderne sein. Dennoch ist die Fähigkeit zu einem solchen staatlichen Handeln Teil der Strukturwirklichkeit moderner Gesellschaften. Und die Modelle, dies zu beschreiben, liegen ja durchaus vor – etwa in der von Claus Offe und Jürgen Habermas schon in den 1970er-Jahren entwickelten Spätkapitalismustheorie, welche die oben genannte Kluft strukturell als Gegensatz zwischen dem auf Gleichheit basierenden demokratischen Prinzip und dem Ungleichheit produzierenden Prinzip privater ökonomischer Aneignung im Staat selbst verortete (Offe 2006; Habermas 1973; vgl. dazu auch Strecker 2013; Borchert und Lessenich 2016). Lessenich selbst hat in den vergangenen Jahren viel dafür getan, eben diese Theorie lebendig und aktuell zu halten. Gewiss sind die von Offe und Habermas identifizierten Strukturprobleme in der Folgezeit nicht so zutage getreten, wie die beiden sie prognostiziert haben, aber in dem aktuell *politisch* sichtbar gewordenen Widerspruch zwischen Systemrelevanz und Lebensrelevanz wird doch offenbar, dass mit den Ansprüchen der Letzteren *strukturelle Handlungsmacht* verknüpft ist.

Es ist jedoch kein Zufall, dass sowohl die herrschaftstheoretisch-strukturdeterministische als auch die systemtheoretische Position diese Möglichkeit des politischen Handelns *gegen die dominanten Strukturlogiken* gar nicht mehr auf der Rechnung haben, dass sie dafür blind geworden sind. Ein konzertiertes Handeln gegen die dominanten gesellschaftlichen Strukturprinzipien und die daraus emanierenden Handlungsimperative ist aus Gründen der gesellschaftlichen *Pfadabhängigkeit* allerdings nur in historischen Ausnahmesituationen möglich und wahrscheinlich. Dass die Corona-Krise einen ebensolchen singulären historischen Ausnahmezustand darstellt, der die Möglichkeit zu einem Pfadwechsel eröffnet, möchte ich im folgenden Abschnitt erläutern.

## Die Krise als singulärer historischer Bifurkationspunkt

Auch wenn die soziologischen Deutungen noch weit auseinandergehen, kann kein Zweifel daran bestehen, dass das globale sozioökonomische System im Frühjahr 2020 eine empfindliche und folgenreiche Störung seiner oftmals weltumspannenden Produktions- und Interaktionsketten, seiner Handlungsroutinen und Entscheidungsregeln und teilweise sogar eine Umkehr seiner Handlungsimperative erlebt hat. Im Zuge der Corona-Krise setzte der Staat die Eigenlogik und -dynamik der Märkte, aber auch des Kultur‑, Bildungs- und Wissenschaftsbetriebs zwar nicht vollständig, aber doch weitgehend außer Kraft und re-etablierte das gesellschaftliche Primat der Politik gegenüber den Grundprinzipien der funktionalen Differenzierung und der Kapitalakkumulation. Wie tiefgreifend dieser Einschnitt ist, zeigt sich auch an der weltweiten Einschränkung elementarer Bürgerrechte wie der Versammlungsfreiheit und an der staatlichen Regulierung des leiblichen Verhaltens auch noch im persönlichen Nahbereich. Indessen bedeutet das partielle Anhalten eines auf dynamischer Stabilisierung beruhenden Systems natürlich noch in keiner Weise seine Neuerfindung oder Neugestaltung; es ähnelt weit eher dem Verursachen eines Unfalls. Die Abbremsung scheint daher mit struktureller Notwendigkeit geradewegs auf einen Systemzusammenbruch hinauszulaufen – jedenfalls dann, wenn sie noch länger andauert und keinem neuen Beschleunigungsschub Platz macht. Das bedeutet, dass es nur die beiden Alternativen zu geben scheint, den gesellschaftlichen Stabilisierungsmodus entweder auf revolutionäre Weise zu verändern oder aber die Dynamisierungsimperative so schnell wie möglich wieder in Kraft zu setzen.

Eben deshalb könnte das Auftreten des Virus einen politisch-sozialen Wendepunkt, den Auftakt zu einem Paradigmenwechsel markieren. Der Grund dafür liegt zum einen in der unerwarteten und – auch und gerade von Soziologen – nicht für möglich gehaltenen Erfahrung kollektiver Selbstwirksamkeit und politischer Handlungsfähigkeit, die sich im Anlegen der Bremsen an die Antriebsräder der dynamischen Stabilisierung gezeigt hat. Nichts kann die handelnden sozialen Akteure *strukturell* daran hindern, diese Erfahrung von Handlungsmacht etwa auf den Umgang mit der Klimakrise oder die schreiende Ungleichheit der Vermögensverhältnisse zu übertragen. Zum anderen liegt der Grund für die Chance auf einen gesellschaftlichen Paradigmenwechsel just in der Krise selbst. Soziopolitische Paradigmenwechsel lassen sich nach dem Modell des Wissenschaftshistorikers Thomas S. Kuhn als Ereignisse verstehen, zu denen es dann kommen kann, wenn ein operatives Paradigma in eine ausgeprägte Krise gerät.^8^ In „normalen“ Zeiten prozessieren die gesellschaftlichen Institutionen und die in ihnen handelnden Akteure entlang festgeschriebener und eingeübter Regeln und Routinen; sie folgen in ihren Problemwahrnehmungen, Aufgabenstellungen und Bearbeitungsmustern festgelegten Pfaden, die so tief verwurzelt sein können, dass ein Wandel selbst beim Auftreten neuartiger Schwierigkeiten fast undenkbar erscheint. In den Sozialwissenschaften hat sich dafür der Begriff der Pfadabhängigkeit eingebürgert: So lange Institutionen und die mit ihnen verbundenen Interaktionsketten halbwegs funktionieren, erscheint der Preis für einen Pfadwechsel zu hoch, und die Risiken, Neues zu versuchen, sind insbesondere angesichts der gewaltigen gesellschaftlichen Komplexität zu groß. In bestimmten Konstellationen aber, zu bestimmten Zeitpunkten im historischen Verlauf, die als geschichtliche „Bifurkationspunkte“ begriffen werden können, eröffnen sich plötzlich Chancen auf einen Pfadwechsel, weil aufgetretene Anomalien nicht mehr ignoriert werden können (vgl. Knöbl 2010; Goldstone 1998). Es handelt sich um Krisenmomente, in denen die Fortsetzung der institutionellen Operationen in Frage steht, in denen eben *nicht* klar ist, wie es weitergeht, weil die Prozessketten gerissen sind. An solchen Gabelungen erscheint es vielen Akteuren wünschenswert, auf den alten Pfad zurückzukehren und so schnell wie möglich die eingespielten Routinen wiederzubeleben. Es ist aber auch möglich, einen neuen Pfad einzuschlagen. Dies sind die seltenen historischen Momente, in denen soziale Akteure Geschichte wirklich *machen* können, in denen es stärker als zu anderen Zeiten auf ihr *Handeln* ankommt, weil sie Momente geschichtlicher Unentschiedenheit und Offenheit sind. Wenn also sowohl Dörre als auch Nassehi betonen, dass die noch so gut gemeinten Intentionen von politischen Aktivist*innen oder Gesellschaftskritiker*innen letztlich notwendig an der Eigengesetzlichkeit systemischer oder materieller Strukturlogiken abprallen (und deshalb Ideologie bleiben müssten), so lautet meine Gegenthese, dass, erstens, das orchestrierte, gesellschaftsgestaltende politische Handeln in der modernen Gesellschaft strukturell sehr wohl angelegt ist und dass es, zweitens, in den Krisenmomenten systemischen Prozessierens wirksam werden kann. Kuhn identifiziert solche Momente als den Ausgangspunkt *wissenschaftlicher* Revolutionen, macht dabei aber deutlich, dass er dieses Modell aus der Beobachtung der *politischen Welt* gewonnen und in die Wissenschaft übertragen hat. Daher ist es nicht verwunderlich, dass das Konzept vom prozesshaften Operieren in „Normalzeiten“ und disruptiven Paradigmenwechseln in Krisenzeiten von Autoren wie Sheldon S. Wolin (1980, 1969), Gary Gutting (1980) oder Joachim Raschke (1980) zur Analyse des gesellschaftlichen Prozessierens wieder in die Sozialwissenschaften zurückübertragen wurde.

Meine zeitdiagnostische These lautet also, dass wir uns angesichts der Stillstellung und Krise zentraler gesellschaftlicher Institutionen an einem solchen historischen Bifurkationspunkt befinden. Hier gibt es keine soziologischen oder ökonomischen Modelle, die vorhersagen könnten, wie es weitergeht. Weil und sofern in dieser Lage die Fortsetzung gesellschaftlicher Prozessketten – und damit: der Fortgang der Geschichte – *tatsächlich offen* ist, ist eine seriöse wissenschaftliche Prognose *konstitutiv ausgeschlossen*. Nicht auf das *Wissen*, sondern auf das *Handeln* kommt es jetzt an. Hier kommt das zum Tragen, was Hans Joas (1992) als die „Kreativität des Handelns“ bezeichnet und Hannah Arendt (1994) als „Natalität“ des Menschen identifiziert hat: Sie meint damit die Fähigkeit, als kreativ handelnde Akteure eingespielte Pfade zu verlassen, geltende Reaktionsweisen und -ketten außer Kraft zu setzen und genuin Neues hervorzubringen. Von einem solchen Neubeginn kann man allerdings gewiss erst dann sprechen, wenn auch die bestehenden Strukturen verändert werden: die Eigentumsstrukturen, die Produktions- und Verteilungsstrukturen, die Konsummuster, die politischen Institutionen. Ein Paradigmenwechsel nur auf der Handlungsebene ist undenkbar.

## Die Rolle der Soziologie am Scheidepunkt

Wenn die These richtig ist, dass es an einem historischen Bifurkationspunkt per se kein wissenschaftliches Modell und keine Theorie geben kann, welche den Fortgang der Entwicklung vorherzusagen vermöchte, und wenn zugleich noch kaum seriöses Wissen über den weiteren Krisenverlauf verfügbar ist – stünde es der Soziologie dann nicht am besten an, sich mit öffentlichen Stellungnahmen und Einschätzungen zurückzuhalten? Solche Zurückhaltung wird derzeit von namhaften Kolleginnen und Kollegen dringend angemahnt. So bemerkt etwa Jürgen Habermas (2020):

Unsere komplexen Gesellschaften begegnen ja ständig großen Unsicherheiten, aber diese treten lokal und ungleichzeitig auf und werden mehr oder weniger unauffällig in dem einen oder anderen Teilsystem der Gesellschaft von den zuständigen Fachleuten abgearbeitet. Demgegenüber verbreitet sich jetzt existentielle Unsicherheit global und gleichzeitig, und zwar in den Köpfen der medial vernetzten Individuen selbst. […] Zudem bezieht sich die Unsicherheit nicht nur auf die Bewältigung der epidemischen Gefahren, sondern auf die völlig unabsehbaren wirtschaftlichen und sozialen Folgen. In dieser Hinsicht – so viel kann man wissen – gibt es, anders als beim Virus, einstweilen keinen Experten, der diese Folgen sicher abschätzen könnte. Die wirtschafts- und sozialwissenschaftlichen Experten sollten sich mit unvorsichtigen Prognosen zurückhalten. Eines kann man sagen: So viel Wissen über unser Nichtwissen und über den Zwang, unter Unsicherheit handeln und leben zu müssen, gab es noch nie.

Interessanterweise scheint er damit just die Diagnose eines gesellschaftlichen Bifurkationspunktes zu bestätigen. Folgt aber aus der Tatsache, dass man nicht *wissen* kann, was jetzt zu tun ist, dass die Soziologie schweigen muss? Meines Erachtens wäre das ein Armutszeugnis insbesondere für jenen Teil der Disziplin, der sich als „public sociology“ versteht, wie sie etwa von Michael Burawoy (2015) und in Deutschland insbesondere auch von Klaus Dörre und Stephan Lessenich vertreten wird. Wenn die Wissenschaft von der Gesellschaft in einer fundamentalen gesellschaftlichen Krise schlicht nichts zu sagen hätte, stellte sich in der Tat die Frage, wozu sie dann taugt und wozu ihre Vertreter*innen bezahlt werden.

Entscheidend für das, was Soziologie in dieser Lage leisten kann, ist die Antwort auf die Frage, als was sie in der Krise auftritt bzw. auftreten will. Mein Vorschlag im Anschluss an Max Weber und Charles Taylor lautet, dass sie sich nicht als Instanz des autoritativen *Wissens*, sondern als Institution der gesellschaftlichen *Selbstdeutung* verstehen sollte. Menschen sind, wie Taylor es formuliert, grundsätzlich selbstinterpretierende Tiere; die (Krisen‑)Lage, in der sich eine Gesellschaft befindet, wird durch den Prozess ihrer kollektiven und diskursiven Deutung stets mitbestimmt. Autoren wie Taylor, Giddens und Habermas haben deshalb für die Sozialwissenschaften den Begriff der „doppelten Hermeneutik“ geprägt: Ihr Kennzeichen ist es, eine zur institutionellen Realität geronnene gesellschaftliche Selbstdeutung mit den begrifflichen Mitteln der Sozialwissenschaften noch einmal zu interpretieren (Taylor 1975; Giddens 1977, S. 12; Habermas 1981, S. 162). Ihre Aufgabe ist es dabei, Vorschläge für einen „best account“, das heißt für die zu einem gegebenen historischen Zeitpunkt bestmögliche Interpretation der gesellschaftlichen Lage zu machen. Wie Manfred Prisching beobachtet hat, entspricht ebendies auch der Erwartung der Menschen an die (öffentliche) Soziologie, insbesondere in Krisenlagen: „Sie wollen angesichts einer unverstehbar gewordenen Gesellschaft ganz einfach wissen, was los ist“ (Prisching 2018, S. 155).^9^

Die Qualität solcher Vorschläge bemisst sich dann aber an ihrer Plausibilität im Lichte aller verfügbarer Daten, Wissensquellen, Erfahrungen und Befunde. Wie Max Weber in seinem leidenschaftlichen Plädoyer für eine engagierte, aber werturteilsfreie Wissenschaft gezeigt hat, steigt diese Qualität insbesondere durch die strikte Verfolgung des Prinzips intellektueller Rechtschaffenheit, das verlangt, vor allem jene Argumente und Befunde ernst zu nehmen und gebührend zu gewichten, die der eigenen Überzeugung und Position widersprechen (Weber 1988; vgl. dazu jetzt auch Müller 2020, S. 58 ff.). Bezogen auf die Corona-Krise bedeutet dies, gleichsam auf hoher See und jederzeit revisionsoffen alles heranzuziehen, was im Lichte des vorhandenen soziologischen Wissens über Gesellschaft dazu beiträgt, mit Hilfe der zur Verfügung stehenden theoretischen Konzepte die Krisenlage und die sich in ihr zeigenden Dynamiken und Entwicklungen zu verstehen. Die so generierten Deutungsvorschläge haben notgedrungen einen Ad-hoc-Charakter: Morgen schon kann eine andere Entwicklung, ein neues Ereignis oder ein neuer Befund die Deutung alt oder falsch erscheinen lassen. Sie verstehen sich, wie gesagt, auch nicht als sakrosanktes Wissen, sondern als das, was sie sind: Interpretationen, die sich im öffentlichen Diskurs der stetigen Kritik nicht nur stellen, sondern geradezu danach streben, durch Einwände und Widersprüche modifiziert und dadurch zu immer besseren „best accounts“ zu werden. Soziologische Analysen tragen auf diese Weise in modernen Gesellschaften zu jenen „accounts“ bei, die schließlich zu wirkmächtigen, sozial operativen gesellschaftlichen Selbstdeutungen werden und in (neuen) Institutionen gerinnen. Soziolog*innen haben dabei gegenüber anderen Bevölkerungsgruppen – auch beispielsweise gegenüber Politiker- und Journalist*innen – den Vorteil, dass ihnen die Ressourcen für die Erfüllung ebendieser Aufgabe von der Gesellschaft selbst zur Verfügung gestellt werden: Sie haben die Zeit, sie haben (zumindest in professoraler Stellung) das sichere Gehalt, sie haben Zugang zu allen Datenbanken und intellektuellen Ressourcen, sie haben das methodische Know-how und die theoretischen Mittel, solche Deutungsvorschläge auf fundierter Basis, im Lichte aller verfügbarer Daten und „der drängenden Kulturprobleme“ zu entwickeln – und darüber hinaus haben sie die nötige Reputation, für ihre Einsichten auch Gehör zu finden. Eine Soziologie, die jetzt schweigt, ist meines Erachtens diese Ressourcen nicht wert.

Zur Entwicklung eines (wenn auch immer nur vorläufigen) „best account“ der gegenwärtigen Krise sind zwei grundsätzliche Fragen zu beantworten: (1) Wohin muss die Soziologie sehen, wenn sie die sich entfaltenden Dynamiken begreifen will? (2) Wie kann sie den sozialen Akteuren helfen, einen kreativen Weg aus der Krise zu finden? Kann und soll sie sich im Lichte denkbarer Alternativen normativ positionieren? Selbstredend kann ich diese Fragen hier nicht erschöpfend diskutieren. Ich möchte aber zu beiden Punkten ein paar Überlegungen anstellen, die in ihrer Gesamtheit das doppelte Ziel verfolgen, erstens zur Formulierung eines aktuellen „best account“ beizutragen und zweitens aufzuzeigen, was die Soziologie aktuell leisten kann und leisten muss.

### Wohin sehen?

Die Corona-Pandemie hat die Gesellschaft weitgehend unvorbereitet und, trotz vereinzelter Warnungen, überraschend getroffen und sie von einem Tag auf den anderen auf allen operativen Ebenen von der Weltpolitik bis in die intimsten Alltagshandlungen hinein massiv beeinflusst und verändert. Eine naheliegende, etwa von Journalisten immer wieder an Soziolog*innen gerichtete Frage lautet daher: *Was passiert gerade mit uns, was passiert mit der Gesellschaft*? Meine eigene erste Antwort darauf, die natürlich durch meine Vorarbeiten präformiert war, lautete: *Wir befinden uns in einem historisch beispiellosen Prozess der Entschleunigung*. Die Reaktionen, die ich daraufhin insbesondere von Kollegen und Kolleginnen erhielt, haben mich überrascht und zum Teil auch irritiert. Die Diagnose der Entschleunigung wurde als politromantische Fantasie eines bestimmten Milieus^10^ oder, noch schlimmer, als rückwärtsgewandte Ideologie, die zu einer neuen „Querfront“ zwischen rechten Demagogen und linken Nostalgikern gegen Emanzipation und Fortschritt führe, diffamiert (Balzer 2020). Die Beschreibung der Lage sei im Ganzen falsch, eine Entschleunigung sei nur für die saturierten Mittel- und Oberschichten gegeben. Klaus Dörre stieß in dasselbe Horn, wenn er mir die „Bauchnabelperspektiven saturierter Milieus“ vorwarf, deren Generalisierung „all jene, die unter den Einschränkungen massiv leiden, [...] wohl nur als zynisch empfinden“ könnten (Dörre 2020c).

Abgesehen davon, dass solche Vorwürfe auf moralische Diffamierung (der reiche Professor denkt nur an sich selbst) und intellektuelle Diskreditierung zielen (der reiche Professor ist auch noch dumm genug, seine eigene Perspektive zu generalisieren), sind sie sachlich schlicht und in einem für den hier zu diskutierenden Punkt höchst signifikanten Sinne falsch. Denn in der Corona-Krise ist Entschleunigung kein normatives Konzept und keine Fantasie, weder eine rechte noch linke, sondern, wie ich bereits dargelegt habe, schlichte Faktizität. Wenn Menschen aus ihren Berufen entlassen und durch Ausgangssperren an ihre Wohnungen gefesselt werden, ist das ganz sicher kein positiver Zustand, aber dennoch eine Form der Stillstellung im Hinblick auf die physische Mobilität und ökonomische Produktivität. Und wenn zahllose Prozessketten im globalen Maßstab angehalten wurden, dann hatte dies eine massive und beispiellose Freisetzung zuvor gebundener Zeitressourcen zur Folge. Auch wenn diese Zeitressourcen dann vielerorts durch neue Aufgaben und Herausforderungen wieder aufgebraucht wurden, führte dies zur verbreiteten Erfahrung einer (wenn auch nur vorübergehenden) Entschleunigung, weil sich die Terminkalender in vielen Milieus sukzessive leerten statt füllten, was für viele Akteure ein substanziell neues Phänomen war. Diese Erfahrung und diese Fakten mit dem Hinweis für irrelevant zu erklären, es gäbe aber auch soziale Lagen oder Milieus, in denen Zeit jetzt knapp werde und vermehrter Stress entstehe (was zweifellos richtig ist, weil die freigesetzten Zeitressourcen sozialstrukturell sehr ungleich verteilt und je nach Position auch sehr unterschiedlich, nämlich als existenzielle Bedrohung oder als wohltuende Pazifizierung, erfahren wurden),^11^ ist ein für eine adäquate Analyse der bestehenden Verhältnisse illegitimer Zug. Er gewinnt instantan die Sympathien des Publikums, weil er Aufmerksamkeit und Sympathie für die gesellschaftlich Vulnerabelsten signalisiert. Diese Aufmerksamkeit ist ganz gewiss gerechtfertigt, und eine Deutung der Corona-Krise, die den zum Teil ungeheuren Leiden derjenigen, die durch sie in eine verschärfte ökonomische, physische oder psychosoziale Not gerieten, keine Rechnung trüge, wäre kein „best account“. Eine Deutung aber, die so sehr auf dieses Leiden fixiert ist, dass sie sich gezwungen sieht, die realen Entschleunigungsprozesse und -phänomene und mit ihnen die Erfahrungen, Wahrnehmungen und Deutungen der anderen sozialen Schichten zu ignorieren^12^ oder gar zu negieren, ist es ebenso wenig. Eine einseitige *normative* Parteinahme für die Benachteiligten lässt sich aus der Sicht mancher soziologischer Ansätze – etwa der Kritischen Theorie, der ich mich selbst zurechne, oder auch der „public sociology“ im Sinne Burawoys – durchaus rechtfertigen. Eine einseitige Verengung der Analyseperspektive auf die Erfahrungswirklichkeit disprivilegierter Schichten mag dagegen rhetorisch billig sein, soziologisch ist sie schlicht unprofessionell: Sie führt, wie ich gleich zeigen werde, zu verzerrten Deutungsvorschlägen, die gerade auf Kosten derjenigen gehen, die man doch zu verteidigen glaubt.

Natürlich wäre eine Deutung der Krise als Entschleunigung darüber hinaus auch dann unvollständig, wenn sie den gleichzeitig manifesten Gegentrend digitaler Beschleunigung unberücksichtigt ließe: Während sich die physisch-materielle Dynamik verlangsamte und der Bewegungsradius vieler Menschen auf einen kleinen Umkreis um ihren Wohnort herum schrumpfte, nahmen die digitalen Veranstaltungen, Produktionen und Interaktionen in vielen Bereichen in hohem Maße zu. Ein „best account“ der Pandemie könnte dies, wie gezeigt, im Sinne der Virilio’schen Vision eines rasenden Stillstandes deuten: Während die physische Mobilität stillgestellt ist, rauschen die Datenströme mit Lichtgeschwindigkeit. Sodann müsste ein struktursensibler soziologischer Deutungsvorschlag der Pandemie dem oben bereits diskutierten Umstand Rechnung tragen, dass sich die Rolle des (National‑)Staates für das gesellschaftliche Leben in der Krise gravierend verändert hat. Die Integration dieser beiden Gesichtspunkte in einen einzigen „best account“ müsste dann wohl von dem Faktum ausgehen, dass nicht das Virus die Bremswirkung erzeugte, sondern erst die (von allen Bevölkerungen mehrheitlich unterstützte) politische Reaktion darauf. Um die motivationalen Ressourcen dieser Reaktion zu verstehen, muss die Soziologie die analytische Perspektive „der dritten Person“, die das gesellschaftliche Geschehen gleichsam „von außen“ auf der Grundlage messbarer Daten beschreibt, mit der kulturwissenschaftlichen Perspektive „der ersten Person“ bzw. mit einer handlungstheoretischen Perspektive verbinden. Mein eigener Vorschlag lautet hier, dass sich jene Reaktion als verzweifelter Versuch der (medizinischen, technischen, politischen) *Verfügbarmachung* des Virus als eines Phänomens deuten lässt, das eine geradezu monströse Unverfügbarkeit symbolisiert und als solche den Albtraum der Moderne verkörpert (Rosa 2020). Ein solcher Versuch eines „best account“ wird niemals vollständig und niemals konkurrenzlos sein können: Gesellschaftliche Selbstdeutungen entwickeln sich im diskursiven Streit heterogenster Perspektiven immer weiter. Für diesen Streit aber argumentative Ressourcen bereitzustellen und daran mitzuwirken, dass die dominanten und schließlich auch wirkmächtig werdenden Deutungen nicht reduktionistisch, unterkomplex oder ideologisch werden – das ist die Aufgabe der Soziologie.

### Wie deuten? Wozu beitragen?

Wenn sich das von Kuhn entwickelte Modell eines Wechsels zwischen langen gesellschaftlichen „Normalphasen“, in denen die Prozessketten operativ geschlossen sind, sodass das Handeln der Akteure festgelegten und habitualisierten Routinen folgt, und tendenziell „revolutionären“ Krisenphasen, in denen jene Regeln plötzlich nicht mehr greifen und habituelle Reaktionsmuster irritiert sind bzw. ins Leere gehen, auf die gegenwärtige Krise anwenden lässt, dann folgt daraus, wie dargelegt, mit zwingender Notwendigkeit, dass man nicht wissen kann, wie es weitergeht – und dass die Art und Weise der Fortsetzung der Prozessketten auch von Zufällen und von dezisionistischen Entscheidungen abhängt. Kuhn selbst spricht für die Situation potenzieller Paradigmenwechsel sogar in der Wissenschaft von Prozessen der „Bekehrung“ und der „Massenüberredung“, die keinen festen Algorithmen mehr folgen (vgl. Kuhn 1989, S. 105 f. und 168). Kurz: Ob die Gesellschaft nach der Pandemie auf den alten Pfad dynamischer Stabilisierung zurückkehrt oder im Sinne der „Natalität“ einen neuen Pfad einschlägt, ist eine Frage des *politischen Handelns*. Dies bedeutet, dass es selbstredend keine soziologische Methode geben kann, den Richtungsstreit zu entscheiden. Was die Soziologie aber sehr wohl leisten kann, ist eine „Wertdiskussion und Wertinterpretation“ im Sinne Max Webers, der die höchste öffentliche Aufgabe der Disziplin darin sah, in einer verwirrenden gesellschaftlichen Lage analytische *Klarheit* über das, was auf dem Spiel steht, und über mögliche Alternativen zu schaffen (Weber 1988, S. 510 ff.).

Konkret auf die aktuelle Lage bezogen, birgt dies für die Soziologie den Auftrag, die Alternative zwischen der Rückkehr zum alten Pfad und dem Wagnis kreativer Neuerfindung herauszuarbeiten – und insbesondere darauf hinzuweisen, dass das vorherrschende operative Paradigma dynamischer Stabilisierung auch schon vor der Corona-Krise in großen Schwierigkeiten steckte und mit einer ganzen Reihe von gravierenden Problemlagen konfrontiert war. Der stetige, über Jahrhunderte hinweg wirkende Zwang zu Wachstum, Beschleunigung und Innovierung hat eine gesellschaftliche Wirklichkeit erzeugt, die auf allen Operationsebenen durch einen Modus der *Aggression* und entsprechende Krisenphänomene gekennzeichnet ist: So lässt sich durchaus konstatieren, dass das dominante steigerungsbasierte Weltverhältnis in der Spätmoderne ein Ausbrennen (burn out) auf der Mikro- und eine Aufheizung (burn up) auf der klimatischen Makroebene sowie der politischen Mesoebene^13^ verursacht. Damit nicht genug: Trotz der stetig steigenden Investition an physischer, politischer und psychischer Energie sind die stabilisierungsnotwendigen Wachstumsraten kaum mehr zu erzielen – zu den ökologischen, demokratischen und psychologischen Krisen der Spätmoderne tritt daher auch pandemieunabhängig eine ausgeprägte ökonomische Krise, die sich u. a. darin äußert, dass selbst negative Zinsen als eine manifeste kapitalistische Anomalie keine ausreichenden Wachstumsimpulse mehr zu setzen vermögen. Eine Rückkehr zum Status quo ante nach dem zu erhoffenden Ende der Corona-Krise scheint daher schlichtweg keine vielversprechende gesellschaftliche Alternative zu sein.

Wenn nun aber namhafte Vertreter*innen des Faches im Brustton tiefster Überzeugung verkünden, dass die Krise eben *keine Chance *zur Veränderung und zur Verbesserung der gesellschaftlichen Verhältnisse insbesondere der Benachteiligten sei, sondern vielmehr eine Verschärfung ihrer Lage zu erwarten stünde, dass aller soziologischen Erkenntnis nach der Kapitalismus nach der Krise noch brutaler, der nationalstaatliche und individuelle Egoismus noch größer, die Verteilungskonflikte noch härter und Solidarität noch unwahrscheinlicher sein werden, dann scheint mir dies analytisch schlicht falsch und normativ-politisch verheerend zu sein. Denn es trägt dazu bei, *in einer gesellschaftlich offenen Situation die bestehenden Verhältnisse zu zementieren und die Rückkehr auf die ausgetretenen Pfade „wissenschaftlich“ zu legitimieren*. Wer so argumentiert, läuft Gefahr – und sei es auch unbeabsichtigt –, das Geschäft der Reifikation zu betreiben, wenn nicht gar der „Reaktion“: Er lähmt alle gesellschaftlichen Impulse, die auf einen Paradigmenwechsel drängen und untergräbt alle Hoffnungen darauf. Er oder sie lässt gerade die Menschen auf der Verliererseite, um die man sich doch angeblich sorgt, im Stich und trägt dazu bei, sie just in einem Moment zu pazifizieren, in dem Veränderung möglich wäre. Vom Elfenbeinturm gesicherter Beamtenverhältnisse aus ergeht die Botschaft: *Geht nach Hause, es gibt keine Chance zur Verbesserung Eures Loses, es wird alles noch viel schlimmer.* Das ist keine „public sociology“, das erscheint mir fast als akademischer Zynismus: Eine als scharfe Kritik formulierte Absage an jede Hoffnung auf Verbesserung befördert die Restauration.

Was Richard J. Bernstein schon 1976 für die Sozialwissenschaften konstatierte, trifft deshalb meines Erachtens im Lichte der Corona-Krise auf die beiden oben skizzierten theoretischen Positionen der Systemtheorie und eines herrschaftstheoretisch-strukturdeterministischen Ansatzes in vollem Umfang zu:

There has been an overwhelming tendency in mainstream social science toward reification, toward mistaking historically conditioned social and political patterns for an unchangeable brute reality which is simply „out there“ to be confronted. […] There has been a tendency to generalize from regularities of a regnant moral paradigm, and to claim we are discovering universal laws […]. The most serious defect in this endeavor is […] the hidden ideological bases […] that has pernicious consequences in limiting human imagination and political and social possibilities (Bernstein 1976, S. 106).

Eine verantwortungsvolle Soziologie im Dienste der Weber’schen Klarheit würde stattdessen auf den durch die Corona-Krise zutage getretenen Doppel-Spalt zwischen Systemrelevanz bzw. ökonomisch erzwungener Steigerungsdynamik einerseits und Reproduktions- oder Lebensrelevanz andererseits sowie zwischen einem globalen Krisenphänomen (dem SARS-CoV-2-Virus) einerseits und den nationalstaatlich limitierten Reaktionen darauf andererseits fokussieren und dadurch die Freisetzung und den Einsatz kreativer gesellschaftlicher Gestaltungsressourcen für einen Paradigmenwechsel befördern. Zu dieser Analyse gehört der Hinweis, dass das dominante Paradigma der Gesellschaft in eine tiefe Krise geraten ist, sodass der operative Normalmodus in vielen Bereichen außer Kraft gesetzt ist – weshalb die Chance auf einen Pfadwechsel *jetzt* gegeben ist. Dass dafür ein wirkmächtiges Instrument in Form des handlungsfähigen Staates zur Verfügung steht, hat die Krisenreaktion eindrucksvoll vor Augen geführt. Erst eine solche Soziologie würde dann indirekt auch dazu beitragen können, das Los der Armen, Unterdrückten und Entrechteten, die unter der Krise am meisten leiden, zu verbessern und sie wirkmächtige politische Subjekte werden zu lassen.

## Footnotes

1 Zwölf eindrucksvolle Kurven solchen exponentiellen Wachstums finden sich bei Bernd Sommer und Harald Welzer (2014, S. 425). Weitere Daten finden sich bei Peter Wagner (2018, S. 43 ff.). Danach ist beispielsweise die Wirtschaftsleistung in den kapitalistischen Ländern des Westens allein zwischen 1820 und 1980 um das 70-fache gewachsen.

2 Das lässt sich sogar messen: So konstatiert eine aufwändige Studie eines internationalen Teams von 76 Wissenschaftlern „the longest and most coherent global seismic noise reduction in recorded history“ (Lecocq et al. 2020, S. 1342) und stellt überdies fest: „A strong correlation between seismic noise and independent measurements of human mobility suggests that seismology provides an absolute, real-time estimate of human activities“ (ebd., S. 1338).

3 Le Quéré et al. (2020, S. 647 f.). Dort findet sich eine Vielzahl weiterer Daten, welche die durchgreifende Entschleunigung in den Sektoren Produktion, Transport und Energieverbrauch eindrucksvoll belegen.

4 Bei einer Forsa-Umfrage vom Sommer 2019 nannten 37 % der Befragten den Umwelt- und Klimaschutz als wichtigstes Thema, 29 % Zuwanderung und die Integration von Flüchtlingen und überraschenderweise nur 13 % Armut und soziale Ungleichheit (Forsa 2019).

5 So hat es der Vermögensbericht von Oxfam (2017) für das Jahr 2016 errechnet. Wenngleich die Berechnungsgrundlage dieser Zahlen im Einzelnen umstritten sein mag, gibt es doch nur wenig Zweifel an der Gesamttendenz. Vgl. dazu natürlich auch weiterhin Thomas Piketty (2016).

6 Ein Beispiel für diesen Hiatus zwischen theoretischer Komplexität und diagnostischem Reduktivismus scheint mir in der Differenz zwischen Lessenichs Theorie des Spätkapitalismus (siehe unten) und seiner eher dependenztheoretischen Analyse der „Externalisierungsgesellschaft“ zu liegen (Lessenich 2016). Ein solcher Reduktivismus liegt meines Erachtens auch dort vor, wo Dörre den Staat einfach auf das strukturelle Ergebnis bzw. die „materielle Verdichtung“ der ungleichen Kräfteverhältnisse zwischen den sozialen Klassen reduziert (Dörre 2020a, S. 31): Es ist genau diese Reduktion, die das staatliche Handeln dann als strukturdeterminiert erscheinen lässt.

7 Marx und Engels haben ihre Konzeption des Basis-Überbau-Verhältnisses und der Rolle des Staates bekanntlich erstmals in der *Deutschen Ideologie *ausbuchstabiert (Marx und Engels 1973).

8 Diese Konzeption von Paradigmen und revolutionären Pfadwechseln hat Kuhn in seinem Buch *Die Struktur wissenschaftlicher Revolutionen* (1989) entwickelt. Zu ihrer Übertragung auf Gesellschaften und Lebensformen siehe Rosa (2012, 1995).

9 Diesen Hinweis verdanke ich Richard A. Brand, der in seiner vorzüglichen Masterarbeit zur „Öffentlichen Soziologie zwischen Autonomie und Engagement“ (FSU Jena 2020) die Möglichkeiten und Grenzen einer „public sociology“ neu ausgelotet hat und dabei von Burawoy zurück zu Weber findet.

10 Armin Nassehi in den ARD-Tagesthemen vom 01.04.2020.

11 Tatsächlich lässt sich zeigen, dass in einer Gesellschaft, die sich nur dynamisch zu stabilisieren vermag, das Ausbleiben ökonomischen Wachstums langfristig die Steigerungs- und Optimierungszwänge und damit auch die stresserzeugenden Faktoren insbesondere für die disprivilegierten Schichten noch erhöht: Entschleunigung ist *innerhalb* solcher Strukturen keine Lösung, sondern ein dysfunktionales Phänomen.

12 Dazu gehört beispielsweise, dass die seit drei Jahren von der „Zeit“ erhobene tägliche Stimmungskurve der Bevölkerung, die sich daran bemisst, dass die Leser*innen per Klick kundtun, ob es ihnen gut geht oder nicht, in der Corona-Krise zum ersten Mal in ihrer Geschichte einen signifikanten positiven Ausschlag anzeigte: Die Anzahl derjenigen, die angaben, es gehe ihnen gut, stieg Mitte März, also just auf dem Höhepunkt der Einschränkungen, von fast invariant konstanten 66 auf mehr als 74 % und flachte danach nur marginal wieder ab. Gewiss sagt dies nur etwas über die Befindlichkeit der „Zeit“-Leser*innen aus – aber diese Befindlichkeit ist analytisch ebenso relevant wie das Unglück anderer Bevölkerungsgruppen (Morasch 2020).

13 Hier beziehe ich mich auf das von dem britischen Politologen Michael Bruter an der London School of Economics geleitete ERC-Projekt „The age of hostility: Understanding the nature, dynamics, determinants, and consequences of citizens’ electoral hostility in 27 democracies“.
